# Variational modeling and numerical simulations for evaporating thin droplets and coffee-ring effect

**DOI:** 10.1140/epje/s10189-026-00603-w

**Published:** 2026-07-06

**Authors:** Yakun Li, Quan Zhao, Tiezheng Qian

**Affiliations:** 1 Department of Mathematics, Hong Kong University of Science and Technology, Clear Water Bay, Kowloon, Hong Kong China; 2 School of Mathematical Sciences, University of Science and Technology of China, Hefei, China

## Abstract

**Abstract:**

Sessile liquid droplets on solid surfaces are ubiquitously found in nature and engineered applications. They exhibit many intriguing properties and phenomena because of the three phases, i.e. liquid, gas, and solid, interacting with each other. Many physical processes are involved in droplet dynamics, presenting a complex problem for fundamental understanding and practical applications. Among those processes participating in the dynamics of evaporating sessile droplets, two have been of continuous interest. The first is the moving contact line at which the evolving liquid–gas interface intersects the solid surface, and the second is the evaporation at liquid–gas interface. Coupled to the moving contact line on the substrate and the liquid flow in the droplet, the interfacial evaporation plays a key role in the evolution of evaporating sessile droplets. Based on Onsager’s variational principle, we derive a continuum model for evaporating thin droplets. The variational approach ensures thermodynamical consistency in describing the coupling of multiple dissipative processes, including viscous momentum transport, contact line motion, evaporation, and vapor diffusion. A characteristic length scale is introduced by considering the competition between the liquid evaporation at liquid–gas interface and the vapor diffusion in gas space. Comparing this intrinsic length scale with that of the confinement geometry, a dimensionless parameter is identified that varies across two distinct dynamic regimes, namely the diffusion-limited regime and the transition-limited regime. Numerical results are presented for the evaporation flux and liquid flow, showing distinct characteristics in these two regimes. The coffee-ring effect is also numerically investigated for drying particle-laden droplets across different dynamic regimes with pinned and depinned contact lines.

**Graphical abstract:**

A characteristic length scale, arising from the competition between evaporation and vapor diffusion, defines a dimensionless parameter that identifies two distinct dynamic regimes. 
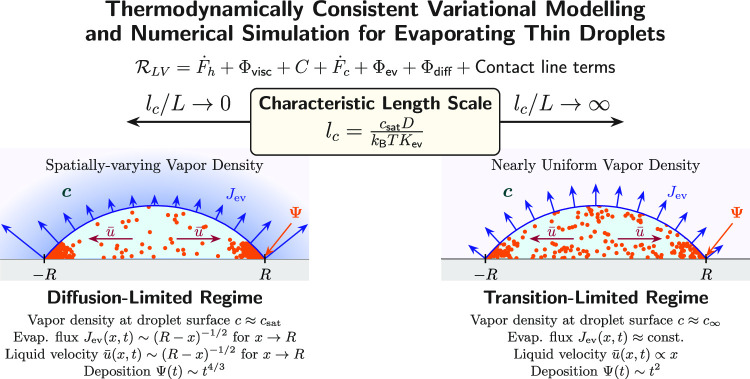

## Introduction

Evaporating droplets [1–4] are ubiquitous in nature and have attracted extensive attention across a wide range of fields covering fundamental research [5–9] and engineering applications, e.g. coating, colloidal assembly, and ink-jet printing [3, 8, 10–14]. The dynamics of evaporating droplets involves multiple transport processes, including viscous momentum transport in liquid, diffusive transport in liquid (for binary-mixture liquid droplets), evaporation at the liquid–gas interface, and diffusion of vapor in gas space. In the presence of three interfaces associated with a sessile droplet, i.e. the liquid–gas, solid–liquid, and solid–gas interfaces, those transport processes are intricately coupled, making the modeling of evaporating droplets a challenging task. Furthermore, the contact line, at which the liquid–gas interface intersects the solid surface, presents an additional challenge. It has been known for decades that a moving contact line is incompatible with the no-slip boundary condition on the solid surface [15–18]. The microscopic dynamics in the three-phase contact line region and its coupling to the liquid evaporation at the liquid–gas interface further complicate the modeling of evaporating sessile droplets.

The phenomena arising from the interplay of hydrodynamic transport, liquid evaporation, vapor diffusion, and contact line motion have garnered significant attention in various fields of study. Among these, the coffee-ring effect stands out as a classic phenomenon, attracting extensive research effort in both fundamental science and practical applications [10, 13, 19–23]. Recently, the active control of deposition patterns has spurred significant interest in the emerging field of dynamic wetting on adaptive, flexible and switchable substrates [24–27]. By applying external stimuli—such as electrowetting, thermal gradients, light-responsive surfaces, and elasto-capillary interactions in soft materials—researchers have been able to actively manipulate the behavior of contact lines and droplets. This capability allows for the direction or suppression of the coffee-ring effect, enabling precise control over how suspended particles are deposited onto the substrate. To effectively capture the underlying physics of these advanced applications, it is essential to develop a comprehensive theoretical framework that integrates complex hydrodynamic transport phenomena with dynamic liquid–solid boundary conditions. Such a framework would help in understanding and predicting the behaviors of liquids on various substrates under different conditions, thus paving the way for innovations in materials science, engineering, and related domains.

Numerous theoretical and computational approaches have been applied to the study of droplet dynamics, including lubrication theory [28–30] for thin films, gradient dynamics models [9, 31], direct numerical simulations [32, 33], diffuse-interface modeling and simulations [34, 35], and many others. Capable of describing the coupling of droplet dynamics, vapor diffusion, and phase transition kinetics, thermodynamically consistent full models are challenging and costly from a computational perspective. To address the computational challenges of full models, reduced-order models have been developed based on Onsager’s variational principle (OVP) [36–40]. By leveraging long-wave approximations and the assumption of slow dynamics (i.e., small capillary number), these models simplify the complexity while maintaining a level of accuracy that is adequate for many applications [9, 22, 31, 41]. This approach enables more efficient simulations while still capturing essential physical phenomena, opening avenues for further exploration in the dynamic wetting landscape.

From a modeling perspective, it is worth pointing out that in the linear response regime [38], OVP [36, 37] has been successfully applied to model the dynamics of droplets on solid surfaces with moving contact lines [18, 22, 42]. Based on this principle, a variety of problems can be formulated—including contact line dynamics [18, 42], thin film evolution under the long wave approximation [31, 41], coupling to other degrees of freedom [31, 39, 40], and reduced-order models [22, 43], with thermodynamic consistency being ensured in the variational framework. In the present work, we extend the variational approach to the modeling of evaporating droplets and carry out analytical and numerical studies for the dynamics of evaporating thin droplets in confined space [9]. Furthermore, in the context of dynamic wetting on adaptive, flexible and switchable substrates [24–27], variational approaches offer significant advantages for modeling the dynamics of droplets on adaptable substrates, particularly in scenarios where wetting energies and friction coefficients vary spatially and temporally through coupling with external fields or other degrees of freedom.

Recent research work has investigated evaporating droplets in two distinct regimes, namely the diffusion-limited regime and the transition-limited regime. The former is controlled by the vapor transport in gas space, while the latter is by the molecular mechanism of evaporation at drop surface [9, 12, 22, 44, 45]. These two regimes are manifested in the spatially varying evaporation flux, which directly affects the liquid flow within the droplet and hence the solid deposition pattern for particle-laden droplets [10, 13, 19–23]. In our variational model, a characteristic length scale can be defined by considering the competition between the liquid evaporation at drop surface and the vapor diffusion in gas space. Comparing this intrinsic length scale with the length scale of confinement geometry, we identify a dimensionless parameter that varies across the two distinct regimes with distinct evaporation fluxes that lead to different deposition patterns of drying particle-laden droplets.

In summary, based on OVP, our thermodynamically consistent model incorporates droplet evolution with contact line motion, vapor diffusion in gas space, and liquid evaporation at droplet surface, with the diffusion-limited regime distinguished from the transition-limited one by a characteristic length scale. We employ a boundary element method (BEM) [46–48] to treat the vapor diffusion in an evolving domain. Our thin film model couples droplet dynamics to vapor diffusion through the liquid evaporation at droplet surface. Computationally, a finite difference method (FDM) is designed to solve the evolution equation for droplet profile, and the BEM to compute the density field for vapor. The coffee-ring effect is also numerically investigated across different dynamic regimes with pinned and depinned contact lines.

The paper is organized as follows. In Sect. 2, we present a detailed model derivation based on OVP. In Sect. 3, we describe the numerical implementation by using the coupled FDM-BEM scheme. In Sect. 4, we provide a series of numerical results to demonstrate how droplet evaporation and deposition pattern are quantitatively modulated by changing three key parameters: (i) the characteristic length scale that distinguishes the diffusion-limited regime from the transition-limited one, (ii) a capillary number that controls the overall speed of drying, and (iii) the friction coefficient that controls the contact line motion. Comparisons with previous works are also made. In Sect. 5, we conclude the paper with a few remarks.

## Variational modeling for evaporating thin droplets

### Onsager’s variational principle

Consider an isothermal system with constant temperature being maintained in both space and time. Its thermodynamic state is described by a set of coarse-grained state variables $\{\alpha _i\}$ with $i=1,\ldots ,n$. In the framework of linear irreversible thermodynamics [38], OVP [36, 37] can be applied as follows [18, 39–41]. Let the rates of change of the state variables $\alpha _i$ be denoted by $\dot{\alpha }_i$. An action functional, commonly called as the Rayleighian [39] and denoted by ${\mathcal {R}}$, can be expressed as

$${\mathcal {R}}=\displaystyle \frac{1}{2}\sum _{i,j=1}^{n}\zeta _{ij}\dot{\alpha }_i\dot{\alpha }_j +\sum _{i=1}^{n}\displaystyle \frac{\partial F}{\partial \alpha _i}\dot{\alpha }_i,$$

in which the right-hand side includes two physically distinct terms. The first term is the dissipation function, hereafter denoted by $\Phi$, which is, by definition, half the rate of free energy dissipation. It is noteworthy that $\Phi$ is quadratic in $\{ \dot{\alpha }_i \}$, and the friction coefficient matrix $\zeta$ is symmetric and positive definite. The second term is the rate of change of the free energy $F=F(\alpha _1,\ldots ,\alpha _n)$, hereafter denoted by $\dot{F}$, which is linear in $\{ \dot{\alpha }_i \}$. Note that the reciprocal symmetry $\zeta _{ij}=\zeta _{ji}$ for the friction coefficient matrix $\zeta$ is a fundamental property that can be derived from the microscopic reversibility [36, 37]. It is based on this symmetry that a variational principle can be formulated to describe the time evolution of the system [36, 37].

Minimizing the Rayleighian $\mathcal {R}$ with respect to the rates $\dot{\alpha }_i$ with $i=1,\ldots ,n$, we obtain

$$\begin{aligned} \sum _{j=1}^{n}\zeta _{ij}\dot{\alpha }_j= -\displaystyle \frac{\partial F}{\partial \alpha _i}, \end{aligned}$$

which expresses the balance between reversible and dissipative forces. The time evolution of the state variables are governed by these equations. It is worth emphasizing that as a result of variation, the rate of free energy dissipation $\dot{F}=\sum _{i=1}^{n}\left( {\partial F}/{\partial \alpha _i}\right) \dot{\alpha }_i$ must equal $-2\Phi$, showing thermodynamic consistency [18, 39–41]. For complex systems that involve many coupled dissipative processes, the application of OVP has proven to be powerful and successful in model derivation.

The dynamics of an evaporating droplet on a solid substrate involves (i) the thin film dynamics of droplet, (ii) the diffusion of vapor, and (iii) the coupling between (i) and (ii) via evaporation at the liquid–gas interface. Below we present an outline of our modular approach to variational modeling. We start from a thin film of an incompressible nonvolatile liquid on a solid substrate [41]. We then allow evaporation to occur at the liquid–gas interface. This introduces a coupling between the dynamics of liquid droplet and the diffusion of vapor which is entropically driven in the gas space.

### Thin film dynamics of nonvolatile liquids

Let us denote the film height profile by $h=h(x,t)$ in a $(2+1)$-dimensional spacetime, with *h* measured in the *z* direction. To derive the thin film hydrodynamics, we consider the free energy functional

1 $$\begin{aligned} F_h[h]=\int \textrm{d}x \, \left\{ \gamma \left[ 1+\frac{1}{2}(\partial _x h)^2\right] +f(h)\right\} , \end{aligned}$$

where $\gamma$ is the liquid–gas interfacial tension, $[1+\frac{1}{2}(\partial _x h)^2]\textrm{d}x$ is the long-wave approximation of the arc length element $\textrm{d}s=[1+(\partial _x h)^2]^{1/2}\textrm{d}x$ along the interface, and *f*(*h*) is the wetting energy locally depending on *h*. The dissipation functional due to shear viscosity is

2 $$\begin{aligned} \Phi _{\text {visc}}=\int \textrm{d}x\int _0^h \textrm{d}z \, \left[ \frac{\eta }{2}(\partial _z u)^2\right] , \end{aligned}$$

where $\eta$ is the shear viscosity and *u* is the *x* component of liquid velocity ${\textbf{v}}(x,z,t)=u(x,z,t)\hat{\textbf{x}}+w(x,z,t)\hat{\textbf{z}}$ in the 2-dimensional *xz* space. For slow variation of *h* with *x*, the rate of viscous dissipation is dominated by $(\partial _z u)^2$ under the long-wave approximation. Finally, there is the constraint term

3 $$\begin{aligned} C=\int \textrm{d}x\int _0^h \textrm{d}z \, \left[ -p(\partial _x u+\partial _z w)\right] , \end{aligned}$$

which imposes the incompressibility condition $\partial _x u+\partial _z w=0$. Here *p* is mathematically a local Lagrange multiplier and physically the local pressure. Note that the general state variables $\{\alpha _i\}$ in Sect. 2.1 correspond to the film height profile *h*(*x*, *t*). Accordingly, the abstract rate variables $\{\dot{\alpha }_i\}$ correspond to the time derivative of the height $\partial _t h$ and the velocity components *u* and *w*, which describe the time evolution of the system.

Using $F_h$, $\Phi _{\text {visc}}$, and *C*, the thin film hydrodynamics can be derived by minimizing the Rayleighian $\mathcal{R}_L=\dot{F}_h + \Phi _{\text {visc}} + C$ with respect to the rates $\partial _t h$, *u*, and *w*. Given the presence of the constraint term *C*, this is a constrained minimization problem in which minimization with respect to the rates will generally lead to the finding of a saddle point of a Lagrangian. Minimizing $\mathcal{R}_L$ with respect to $\partial _t h$, we obtain $p(x,h,t)=-\gamma \partial _x^2 h+f'(h)$ for the pressure at $z=h$. Minimizing $\mathcal{R}_L$ with respect to *u* and *w*, we obtain ${\eta }\partial _z^2 u - \partial _x p=0$ for the force balance in the *x* direction and $\partial _z p=0$ for the hydrostatic condition in the *z* direction, respectively. In addition, minimizing $\mathcal{R}_L$ with respect to *u* at $z=h$, we have ${\eta }\partial _z u=0$ for the tangential stress at the liquid–gas interface. Combining the above results with the no-slip condition $u=0$ at $z=0$ (though we understand that the Navier slip condition can be applied as well [18]), we can obtain a parabolic profile for *u*(*x*, *z*, *t*):

4 $$\begin{aligned} u(x,z,t)=\displaystyle \frac{1}{\eta } \partial _x \left[ -\gamma \partial _x^2 h+f'(h) \right] \left( \displaystyle \frac{z^2}{2}-zh\right) , \end{aligned}$$

and the corresponding flux $J_h=\int _0^h \textrm{d}z\, u$ given by $J_h=\displaystyle \frac{h^3}{3\eta } \partial _x \left[ \gamma \partial _x^2\,h-f'(h) \right]$. Note that due to a separation of macroscopic and microscopic length scales, the no-slip condition used in deriving Eq. (4) can coexist with a moving contact line that exhibits slip in a microscopic region. Substituting $J_h$ into the continuity equation $\partial _t h=-\partial _x J_h$, we obtain the thin film evolution equation

5 $$\begin{aligned} \partial _t h = -\partial _x \left\{ \displaystyle \frac{h^3}{3\eta } \partial _x \left[ \gamma \partial _x^2 h-f'(h) \right] \right\} . \end{aligned}$$

The variational approach ensures thermodynamic consistency, and Eq. (5) can be cast in the form of the gradient dynamics [31, 41]:

6 $$\begin{aligned} \partial _t h =-\partial _x \left[ \displaystyle \frac{h^3}{3\eta } \left( -\partial _x \displaystyle \frac{\delta F_h}{\delta h} \right) \right] , \end{aligned}$$

with the variational derivative ${\delta }F_h/{\delta h}=-\gamma \partial _x^2 h+f'(h)$. Note that before evaporation is incorporated, *h* is a conserved field.

### Dynamics of evaporating thin droplets

In the presence of evaporation at the liquid–gas interface, we have the kinematic equation

7 $$\begin{aligned} v_\perp =U+J_{\text {ev}}/N_L. \end{aligned}$$

Here $v_\perp$ is the liquid velocity component normal to the interface and pointing from the liquid into the vapor, given by $v_\perp \approx w - u \partial _x h$ for small $|\partial _x h|$, *U* is the interfacial velocity normal to the interface, $J_{\text {ev}}$ is the evaporation flux, and $N_L$ is the number density of liquid particles. Physically, Eq. (7) a kinematic condition necessitated by mass conservation: the liquid flow normal to the interface is used for both the interface movement and the liquid-to-vapor transition. The free energy functional associated with vapor distribution is of entropic origin and given by

8 $$\begin{aligned} F_c[c]=k_{\text{ B }}T\iint _{\Omega _g} \textrm{d}x \textrm{d}z \, c \log \frac{c}{c_0} , \end{aligned}$$

where $k_\text {B}$ is the Boltzmann constant, *T* is the temperature, ${\Omega _g}$ denotes the gas space, $c=c(x,z,t)$ is the number density of vapor, and $c_0$ is a reference density. The dissipation functionals associated with evaporation and vapor diffusion are, respectively, given by

9 $$\begin{aligned} \Phi _{\text {ev}}=\int _{\Gamma _{lg}} \textrm{d}s \, \frac{J_{\text {ev}}^2}{2K_{\text {ev}}}, \quad \Phi _{\text {diff}}=\iint _{\Omega _g} \textrm{d}x \textrm{d}z \, \frac{\zeta \textbf{j}^2 }{2c} , \end{aligned}$$

where $\Gamma _{lg}$ denotes the liquid–gas interface, $K_{\text {ev}}$ is a kinetic coefficient controlling the rate of evaporation, $\zeta$ is the drag coefficient for vapor particles in air, and $\textbf{j}$ is the diffusion current density. To describe the thin film hydrodynamics coupled with vapor diffusion via evaporation at the liquid–gas interface, we construct the total Rayleighian $\mathcal{R}_{LV}=\mathcal{R}_L+\dot{F}_c+\Phi _{\text {ev}}+\Phi _{\text {diff}}$, in which $\mathcal{R}_L$, given by $\mathcal{R}_L=\dot{F}_h + \Phi _{\text {visc}} + C$, has been shown to describe the thin film dynamics of liquids in Sect. 2.2. With the help of Eq. (7), we first minimize $\mathcal{R}_{LV}$ with respect to the rates $\partial _t h$, *u*, and *w* to obtain the pressure *p*(*x*, *h*, *t*) and the parabolic profile for *u*(*x*, *z*, *t*), which are the *same* as those derived in Sect. 2.2. We then minimize $\mathcal{R}_{LV}$ with respect to the rates $J_{\text {ev}}$ and $\textbf{j}$ to obtain two constitutive equations: $J_{\text {ev}}=K_{\text {ev}} \left( \frac{1}{N_L}p - \mu _c\right)$ for evaporation at the interface and $\textbf{j}=-\frac{c}{\zeta }\nabla \mu _c=-\frac{k_{\text{ B }}T}{\zeta }\nabla c$ for vapor diffusion, where $\mu _c={\delta }F_c/{\delta c}=k_{\text{ B }}T \left[ \log ({c}/{c_0})+1 \right]$ is the chemical potential of vapor.

Here we give some details on the derivation of $J_{\text {ev}}$, which is the key quantity linking the droplet dynamics to vapor diffusion. Firstly, the constraint term $C=\int \textrm{d}x \int _0^h \textrm{d}z \left[ -p\nabla \cdot \textbf{v}\right]$ can be written as

10 $$\begin{aligned} C=\int \textrm{d}x \int _0^h \textrm{d}z\, \nabla p \cdot \textbf{v}+\int _{\Gamma _{lg}} \textrm{d}s\, \left[ -p \left( U+\frac{J_{\text {ev}}}{N_L}\right) \right] , \end{aligned}$$

through an integration by parts, with Eq. (7) used for $v_\perp$ normal to the interface. Secondly, the rate of change of the free energy $F_c$, i.e. $\dot{F}_{c} =\iint _{\Omega _g} \textrm{d}x \textrm{d}z \, \mu _c \partial _t c$, is found to be

11 $$\begin{aligned} \dot{F}_{c} =\iint _{\Omega _g} \textrm{d}x \textrm{d}z \, \nabla \mu _c \cdot \textbf{j}+\int _{\Gamma _{lg}} \textrm{d}s \,\mu _c J_{\text {ev}}, \end{aligned}$$

where the continuity equation $\partial _t c=-\nabla \cdot \textbf{j}$ is used, an integration by parts is involved, and $J_{\text {ev}}$ gives the normal component of the diffusion current density $\textbf{j}$ at the interface, i.e. $J_{\text {ev}}=-\textbf{n}\cdot \textbf{j}=D\partial _\textbf{n} c$, with $D={k_{\text{ B }}T}/{\zeta }$ being the diffusion coefficient and $\textbf{n}$ denoting the unit normal vector pointing from the vapor into the liquid. Thirdly, there is the dissipation functional $\Phi _{\text {ev}}$ quadratic in $J_{\text {ev}}$. Finally, to minimize $\mathcal{R}_{LV}$ with respect to $J_{\text {ev}}$, we need $C+\dot{F}_{c}+\Phi _{\text {ev}}$, from which the constitutive equation for $J_{\text {ev}}$ can be readily obtained.

The variational modeling outlined above leads to the following governing equations

12a $$\begin{aligned}&\partial _t h = -\partial _x \left( \displaystyle \frac{\gamma h^3}{3\eta } \partial _x^3 h \right) -v_m J_{\text {ev}}, \end{aligned}$$

12b $$\begin{aligned}&\partial _t c = -\nabla \cdot \textbf{j} = D\nabla ^2 c,\;\; \text {with } \textbf{j}=-D \nabla c, \end{aligned}$$

12c $$\begin{aligned}&J_{\text {ev}} =K_{\text {ev}} \left( -v_m \gamma \partial _x^2 h + k_{\text{ B }}T \log \frac{c_{\text {sat}}}{c}\right) , \end{aligned}$$

where $v_m=1/N_L$ is the molecular volume of liquid particles, and $c_{\text {sat}}$ is the saturated vapor density for non-curved interface. The three terms in Eq. (12c) are from $\Phi _{\text {ev}}$ in Eq. (9), *C* in Eq. (10), and $\dot{F}_{c}$ in Eq. (11), respectively. For simplicity, we have taken $f'(h)=\text {const.}$, which determines $c_{\text {sat}}$ through $v_m f'(h)=\mu _c(c_{\text {sat}})$. This leads to $v_m f'(h)-\mu _c(c)= k_{\text{ B }}T \log ({c_{\text {sat}}}/{c})$ in Eq. (12c). Here we note the following: (i) $J_{\text {ev}}$ depends on the liquid–gas interfacial curvature $-\partial _x^2 h$ and the vapor density *c* at the interface. (ii) For mass conservation, $J_{\text {ev}}$ equals the normal component of the diffusion current density $\textbf{j}$ at the interface, i.e. $J_{\text {ev}}=D\partial _\textbf{n} c$. (iii) Equation (12a) for *h* and Eq. (12b) for *c* are coupled through (i) and (ii). (iv) $J_{\text {ev}}$ in Eq. (12c) leads to the Ostwald–Freundlich equation [49]. To make $J_{\text {ev}}=0$ at saturation, *c* is greater than $c_{\text {sat}}$ due to the curvature $-\partial _x^2 h>0$, i.e., the saturation vapor density is greater than $c_{\text {sat}}$ for non-curved interface due to the capillary pressure $-\gamma \partial _x^2 h>0$. Here the vapor diffusion is considered as a slow process in the gas space, where the mixture of air and vapor maintains a constant pressure via fast mechanical equilibration.

In general, the variational approach based on OVP allows the model to be further generalized in many regards by introducing more realistic free energy and dissipation functionals, with thermodynamic consistency being ensured in coupling multiple dissipative processes.

## Numerical method

**Fig. 1:**
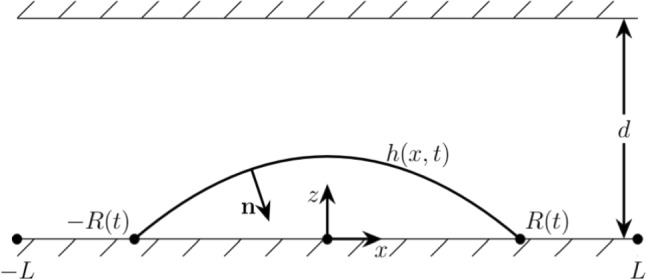
A schematic illustration for a liquid drop in the *xz* plane. The height profile *h*(*x*, *t*) is defined for $x \in [-R(t), R(t)]$, with the two contact lines at $x=\pm R(t)$. The computational domain is $[-L, L] \times [0, d]$, where *d* denotes the distance between the bottom substrate and the top plate. The unit normal vector $\textbf{n}$ points from the vapor into the liquid at the interface

In our numerical simulations, the thin film evolution is governed by Eq. (12a) supplemented with Eq. (12c). As to the vapor diffusion described by Eq. (12b), the vapor density *c* is assumed to adjust swiftly compared to the droplet drying and interface evolution, and hence *c* satisfies the Laplace equation $\nabla ^2 c=0$ for quasi-steady state [10, 12]. A schematic illustration is given in Fig. 1.

Symmetric about $x=0$, the droplet ends at the two contact lines at $x=\pm R(t)$, i.e. $h(\pm {R}(t),t)=0$. The contact line velocity $\dot{R}$ is governed by the constitutive equation

13 $$\begin{aligned} \beta \dot{R}=\gamma (\cos \theta _e-\cos \theta _d)=\frac{\gamma }{2}\left[ \left( \partial _x h\right) ^2 - \theta _e^2 \right] , \end{aligned}$$

where $\theta _e$ is the equilibrium contact angle, and $\theta _d$ the dynamic contact angle, with $\cos \theta _e\approx 1-\theta _e^2/2$ and $\theta _d\approx |\partial _xh|$. Equation (13) can be derived within the framework presented in Sects. 2.2 and 2.3. This makes use of $(1/2)\beta \dot{R}^2$ as the dissipation function due to the contact line friction, which is localized in the microscopic contact line region where the no-slip condition breaks down. In relation to Eq. (13), there have been previous works on the modeling and simulation of the dynamic contact angle [50–52]. The effective contact line dissipation generally consists of two contributions: a macroscopic hydrodynamic dissipation within a wedge (which is due to liquid viscosity and scales as $1/\theta _d$ [53]) and a microscopic friction modeled by the friction coefficient $\beta$ here. Necessitated by the formation of ring-like deposition patterns, the pinning or slow motion of the contact line can be effectively achieved by using a sufficiently large value for $\beta$, without explicitly including the hydrodynamic viscous dissipation $\sim 1/\theta _d$.

Combining Eq. (12c) for $J_{\text {ev}}$ and the condition $J_{\text {ev}}=D\partial _{\textbf {n}} c$ at the interface, we obtain the interfacial condition for the vapor density *c*:

14 $$\begin{aligned} l_c \partial _\textbf{n} c=c_{\text {sat}}\left[ - \Lambda \partial _x^2 h + \log ({c_\text {sat}}/{c})\right] , \end{aligned}$$

where the characteristic length $l_c={ c_{\text {sat}} D}/({k_{\text {B}} T K_{\text {ev}}})$ is defined from the competition between diffusion (controlled by *D*) and evaporation (controlled by $K_{\text {ev}}$), and the length scale $\Lambda ={v_m \gamma }/({k_{\text {B}} T})$ is of nanometer scale, physically too small for the curvature $\partial _x^2 h$ to take effect. Therefore, we simplify Eq. (14) and use

15 $$\begin{aligned} l_c \partial _\textbf{n} c= c_{\text {sat}} \log ({c_\text {sat}}/{c}) \end{aligned}$$

at the interface where droplet evolution is coupled with vapor diffusion.

Following the above simplification, the whole system becomes

16 $$\begin{aligned} \left\{ \begin{array}{ll} \partial _t h =-\partial _x \left( \frac{\gamma h^3}{3\eta } \partial _x^3 h \right) - v_m D \partial _\textbf{n} c \; &  \text{ in } \; [-R, R], \\ \nabla ^2 c=0 \; &  \text{ in } \; \Omega _g,\\ \beta \dot{R}= \frac{\gamma }{2} [(\partial _x h)^2-\theta _e^2] \; &  \text{ at } \; x=\pm R,\\ l_c \partial _\textbf{n} c= c_{\text {sat}} \log ({c_\text {sat}}/{c}) \; &  \text{ on } \; \Gamma _{lg}, \end{array} \right. \end{aligned}$$

supplemented with the boundary conditions given by $h(\pm {R}(t),t)=0$ and

17 $$\begin{aligned} \partial _z c|_{z=d}=0,\;\partial _z c|_{x>|R|,z=0}=0,\; c|_{x=\pm L}=c_{\infty }. \end{aligned}$$

Here the no-flux condition is applied at the top plate and at the solid–gas interface ($x>|R|$ up to the left or right boundary of the domain), and the ambient vapor density $c_\infty$ is imposed at the left and right boundaries $x=\mp L$. In Eq. (16), the thin film evolution equation is a classic degenerate parabolic equation. At the moving boundary, i.e., the contact line, we have $h=0$, which makes the mobility ($\propto h^3$) vanish toward the contact line. This causes the equation to degenerate, and this degeneracy intrinsically ensures that the mass flux naturally vanishes at the boundary of $h=0$. Therefore, no additional boundary conditions for higher-order derivatives of *h* are needed [50].

To nondimensionalize the above system, we use $\mathcal{L}=R(0)$ as the length unit and $\frac{\gamma }{\eta }$ as the velocity unit. Accordingly, $\tau ={\eta }\mathcal{L}/{\gamma }$ is the time unit, $\overline{h}={h}/\mathcal{L}$ is the dimensionless height, $\overline{c}=c/c_\text {sat}$ is the dimensionless vapor density, $\overline{l}_c={l_c}/\mathcal{L}$ is the dimensionless characteristic length corresponding to $l_c$, ${\textrm{Ca}} ={\eta } v_m D c_\text {sat}/{\gamma }\mathcal{L}$ is a dimensionless capillary number with $v_m D c_\text {sat}/\mathcal{L}$ being a velocity scale, and $\overline{\beta }={\beta }/{\eta }$ is the dimensionless contact line friction coefficient. Dropping the bar over the dimensionless quantities, we have

18 $$\begin{aligned} \left\{ \begin{aligned}&\partial _t h =-({1}/{3})\partial _x \left( h^3 \partial _x^3 h \right) - {\textrm{Ca}} \partial _\textbf{n} c,\\&\nabla ^2 c=0,\\&\beta \dot{R}= (1/2)[(\partial _x h)^2-\theta _e^2],\\&{l_c} \partial _\textbf{n} c=\log ({{1}/{c}}). \end{aligned} \right. \end{aligned}$$

Regarding Eqs. (16) and (18), we point out that a small slip length will be effectively introduced as follows. By mapping the system onto a computational domain for numerical solution in discretized space, the no-slip condition effectively breaks down at a cut-off length scale, i.e., the grid size. This regularizes the divergence of viscous dissipation caused by the inconsistency between the no-slip condition and the moving contact line. The contact-line slip actually occurs in a microscopic region that is smaller than or comparable to the cut-off length scale. In this sense, Eqs. (16) and (18) present a complementary description of droplet dynamics from the bulk to the contact line. The first equation in (18), featuring the $h^3$ mobility, governs the macroscopic bulk region where the no-slip condition holds up to the cut-off length scale. The no-slip condition breaks down at the cut-off length scale where the contact-line slip occurs, with the local rate of free-energy dissipation modeled by the friction coefficient $\beta$ in the third equation. See Appendix A for a discussion on the quantitative effects of $\beta$ and the grid size.

For droplet evolution coupled with vapor diffusion through the evaporation at the interface, the length scale $l_c$ plays a critical role when the coupled dynamics occurs in a space that exhibits its own length scale, e.g., *R*, *d*, or *L*. A comparison between these two length scales leads to two distinct dynamic regimes. In terms of the dimensionless characteristic length $l_c$, the diffusion-limited regime corresponds to $l_c\rightarrow 0$, which implies $c\approx 1$ at the interface. This leads to ${l_c} \partial _\textbf{n} c=\log ({{1}/{c}}) \approx 1-c$, with the dimensionless saturation density given by 1. For sufficiently large $l_c$, the system enters into the transition-limited regime with the dimensionless $c\approx c_\infty /c_\text {sat}$ at the interface, and hence ${l_c} \partial _\textbf{n} c\approx \log ( c_{\text {sat}}/c_{\infty })=\text {const.}$ corresponding to a homogeneous evaporation flux $J_{\text {ev}}$.

To solve the above system numerically, for the thin film equation, we rescale the variables and parameters and use the coordinate transformation $x(\xi ,t)=R\xi$ to map the time-dependent domain $[-R(t),R(t)]$ to the fixed computational domain $[-1,1]$. In terms of $\xi$ and *t*, the thin film equation reads

19 $$\begin{aligned} \frac{\partial h}{\partial t} = \frac{x_t}{x_\xi } \frac{\partial h}{\partial \xi }-\frac{1}{x_\xi } \frac{\partial }{\partial \xi } \left( \frac{ h^3}{3} \frac{1}{x_\xi ^3} \frac{\partial ^3 h}{\partial \xi ^3} \right) -{\textrm{Ca}} \partial _{\textbf{n}} c, \end{aligned}$$

with $x_t=\dot{R}\xi$ and $x_\xi =R$. To solve Eq. (19), the computational domain $[-1,1]$ is divided into *N* grids of equal size $\Delta \xi =2/N$, with the droplet height *h* evaluated at the half-grid points using a second-order FDM. Concurrently, the Laplace equation for *c* is to be solved in the gas space which is an irregular computational domain determined by the evolving droplet profile. In this regard, the BEM is suitable for spatial discretization. At each time step, the BEM is first employed to solve for the density field *c*, thereby yielding the updated evaporation flux. This flux is then incorporated into the FDM to advance the height profile *h* via Eq. (19). Boundary element mesh reconstruction is employed when the contact line distortion exceeds a predetermined threshold. A first-order explicit Euler scheme is employed for computing time evolution (see Appendix A for details).

To numerically investigate the coffee-ring effect for droplets of colloidal suspension, we assume that (i) the colloidal particles are driven by the liquid velocity as long as they remain in the droplet, and (ii) the variation of the volume fraction of colloidal particles $\varphi (x,z,t)$ is negligibly small in the *z* direction, i.e., $\varphi (x,z,t)\approx \phi (x,t)$. We have $\partial _t \psi + \partial _x \left( \psi \bar{u}\right) =0$, where $\psi (x,t)=h(x,t) \phi (x,t)$ is the effective height of colloidal particles, and $\bar{u}=\frac{1}{3} h^2 \partial _x^3 h$ is the height-averaged liquid velocity. Instead of solving the above continuity equation for $\psi (x,t)$, we can also use $\bar{u}$ to directly compute the motion of colloidal particles [22]. Driven by the out-going liquid flow, particles are deposited onto the substrate when they reach the contact line and remain fixed afterward.

## Numerical results and discussion

The numerical results presented in this section use the dimensionless computational domain $[-10,10]\times [0,1.2]$ and the dimensionless ambient vapor density fixed at $c_{\infty }=0.1$. Three representative values of the characteristic length $l_c$ are used to access different evaporation regimes: $l_c=10^{-3}$ for the diffusion-limited regime, $l_c=2$ for the intermediate regime, and $l_c=10^3$ for the transition-limited regime.

### Evaporation of thin droplets: the two distinct regimes

**Fig. 2:**
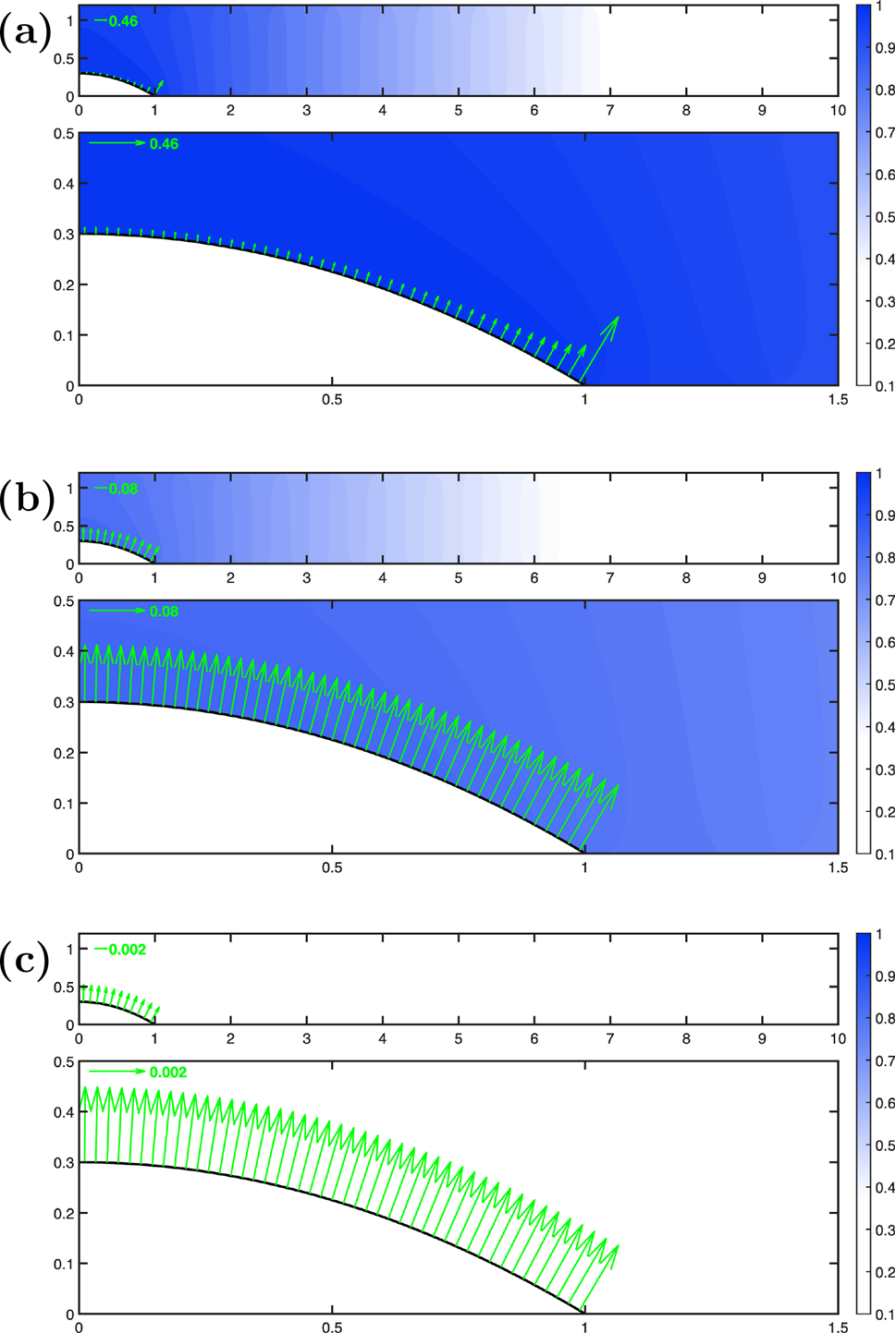
Vapor density *c* and evaporation flux $\partial _\textbf{n} c$ computed in three different regimes using different values of $l_c$. (a) Diffusion-limited regime ($l_c=10^{-3}$) showing large density gradient in gas space and diverging evaporation flux near the contact line. (b) Intermediate regime ($l_c=2$) showing moderate behaviors. (c) Transition-limited regime ($l_c=10^3$) showing nearly uniform density field and almost homogeneous evaporation flux

We first show the vapor density field and evaporation flux distribution, with the contact line being pinned and the characteristic length scale $l_c$ being varied from the diffusion-limited to the transition-limited regime [9]. Figure 2 shows the diffusion-limited, intermediate, and transition-limited regimes computed using $l_c=10^{-3}$, $2$, and $10^3$, respectively. It is observed that an increasing $l_c$ leads to a clear change in the vapor density field and evaporation flux distribution. For $l_c=10^{-3}$, the density field shows a visible variation in the *x* direction from $c\approx c_\text {sat}$ near the droplet to $c\approx c_{\infty }$ near the boundary. Furthermore, the evaporation flux rapidly increases in approaching the contact line, the characteristic of the diffusion-limited regime where the vapor diffusion is the slow process limiting the droplet drying. As $l_c$ becomes large enough, the system enters into the transition-limited regime where the vapor density is notably uniform due to fast diffusion, and the droplet drying is limited by the phase change kinetics at droplet surface where the evaporation flux is almost homogeneous. These observations are consistent with the simulation results obtained from the Stokes description in Ref. [9].

**Fig. 3:**
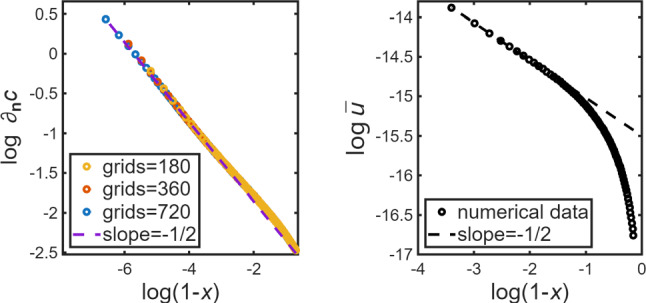
Log-log plot for the evaporation flux $\partial _\textbf{n} c$ and the height-averaged liquid velocity $\bar{u}$ near the contact line ($x=1$) in the diffusion-limited regime. Both quantities show a power law $\sim (1-x)^{-1/2}$

The diffusion-limited regime is validated with numerical accuracy by showing the evaporation flux $\partial _\textbf{n} c$ and the height-averaged liquid velocity $\bar{u}$ near the contact line in Fig. 3. Both quantities tend to diverge near the contact line, exhibiting a power law of exponent $-1/2$. This confirms the theoretical result and experimental observation for small contact angle [10, 12, 54]. Note that the power law $\sim (1-x)^{-1/2}$ is an asymptotic behavior that is valid only in a very small region near the contact line. In the right panel of Fig. 3, this power law shows up in the region close to the contact line. Away from the contact line, the velocity $\bar{u}$ naturally deviates from the power law.

### Coffee-ring effect with pinned contact lines

**Fig. 4:**
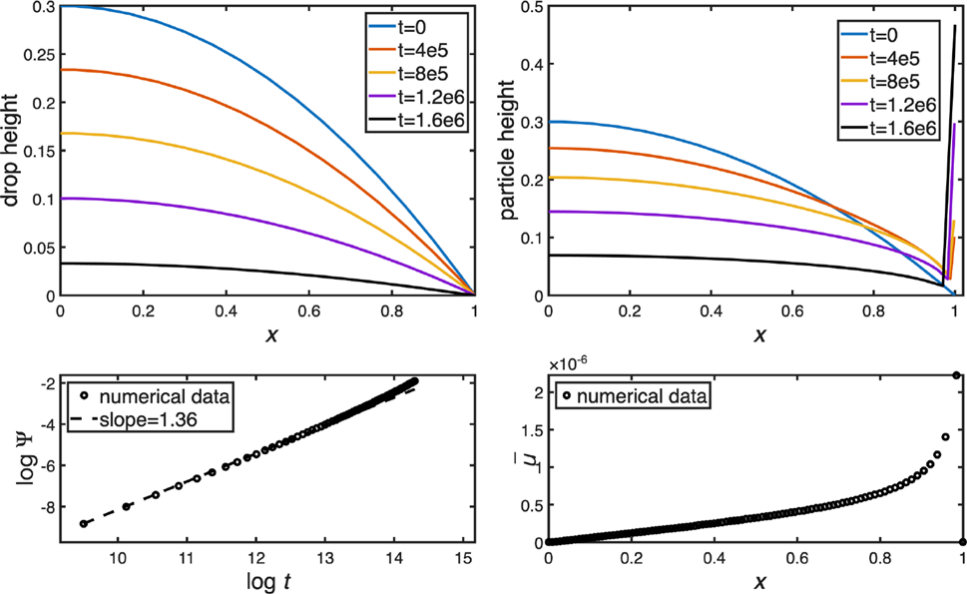
Temporal evolution of droplet profile (top left) and particle height (top right) in the diffusion-limited regime with $l_c=10^{-3}$ and ${\textrm{Ca}}=10^{-6}$. The power law $\Psi \sim t^{4/3}$ is shown in a log-log plot (low left), and the height-averaged liquid velocity $\bar{u}$, plotted for $t=10^6$, tends to diverge as $x\rightarrow 1$ (low right)

Figure 4 shows the temporal evolution of droplet profile and particle height in the diffusion-limited regime. Here the particle height $\psi (x,t)=h(x,t) \phi (x,t)$ is scaled to make $\psi (0,0)=h(0,0)$ by setting $\phi (x,0)=1$. The droplet volume decreases linearly with time. This is theoretically expected and agrees with the numerical finding in Ref. [9]. The particles accumulate at the pinned contact line, leading to the formation of ring pattern reported in Ref. [10]. The log-log plot for the deposited amount of solid $\Psi$ as a function of time agrees with the power-law scaling $\Psi \sim t^{4/3}$ observed in experiments [10]. This is caused by the evaporation flux and the height-averaged liquid velocity that tend to diverge near the contact line, as shown in Figs. 3 and 4 (see Appendix B).

**Fig. 5:**
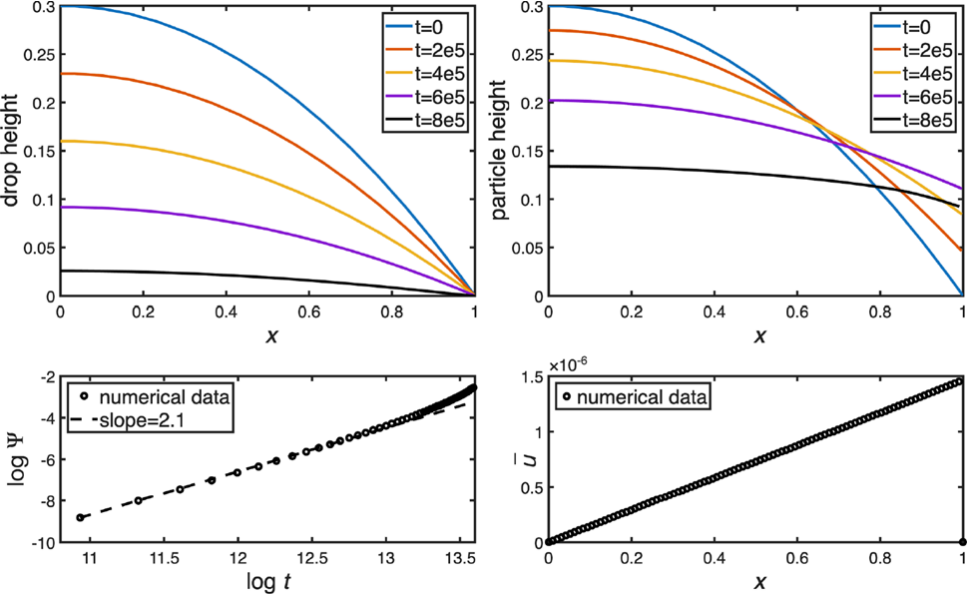
Temporal evolution of droplet profile (top left) and particle height (top right) in the transition-limited regime with $l_c=10^3$ and ${\textrm{Ca}}=10^{-4}$. The power law $\Psi \sim t^{2}$ is shown in a log-log plot (low left), and the height-averaged liquid velocity $\bar{u}$, plotted for $t=6\times 10^5$, is linear in *x* (low right)

Figure 5 shows the temporal evolution of droplet profile and particle height in the transition-limited regime. The droplet volume still decreases linearly with time. The homogeneous evaporation flux leads to a velocity field with $\bar{u}(x,t)\propto x$. Without a diverging $\bar{u}$ for $x\rightarrow 1$, the particle accumulation in the course of time differs significantly from that in Fig. 4, showing a new power-law scaling $\Psi \sim t^2$ (see Appendix B).

In summary, Fig. 2 shows that there are two distinct regimes for droplet evaporation in confined space, and Fig. 3 presents the diverging behavior of the evaporation flux and liquid velocity near the contact line in the diffusion-limited regime. More distinctions between the two regimes can be found in Figs. 4 and 5. It is noted that in the late stage of drying, the droplet can no longer maintain a parabolic profile. This deviation may be attributed to the liquid velocity that is enhanced by the diminishing droplet volume while the evaporation flux remains constant in time.

### Coffee-ring effect with depinned contact lines

In the asymptotic regime where evaporation proceeds slowly, the evolving droplet retains a parabolic shape, and its state is fully described by two state variables, i.e. the volume *V* and the contact line position *R*. Given the control parameters $(\textrm{Ca}, \beta )$, an infinitesimal development from one state $\mathcal{S}_1$ to the next state $\mathcal{S}_2$ takes a time step $\Delta t$. If $(\textrm{Ca}, \beta )$ are scaled to $(m\times \textrm{Ca}, \beta /m)$, then the rates of the two state variables, i.e. $\dot{V}$ and $\dot{R}$, are amplified by a factor of *m*, and hence the time step becomes $\Delta t/m$ for the infinitesimal development of state from $\mathcal{S}_1$ to $\mathcal{S}_2$. This scaling follows from Eq. (18) with the evaporation term $\propto \textrm{Ca}$ and $\dot{R} \propto \beta ^{-1}$. Therefore, $\dot{V}$ and $\dot{R}$ are determined by the rate coefficients $\textrm{Ca}$ and $\beta ^{-1}$ and the instantaneous droplet profile. This argument is to be quantitatively verified in the two distinct regimes below. In our simulations, the equilibrium contact angle $\theta _e$ is set to be the initial contact angle, i.e., $|\partial _x h|$ at $x=1$ and $t=0$. This ensures that the droplet is initially in mechanical equilibrium. As a result, the subsequent contact line motion is entirely driven by the continuous evaporation and consequently the evolution of height profile.

#### Diffusion-limited regime

The diffusion-limited regime with depinned contact lines remains an open and largely unexplored problem for particle deposition. In contrast to the transition-limited regime characterized by homogeneous evaporation flux and hence a linear liquid velocity [22], the diffusion-limited regime features spatially fast-varying evaporation flux and liquid velocity that tend to diverge near the contact line. Such extreme spatial inhomogeneity complicates any direct application of theories developed for homogeneous flux [22]. In the diffusion-limited regime, the pioneering work by Deegan et al. [10] focused on pinned contact lines without considering the effect of contact line motion on evaporation-induced particle deposition. Moreover, Hartmann et al. [9] investigated vapor and liquid dynamics in both regimes without dealing with solid particle transport and deposition. Here we use $l_c=10^{-3}$ for this regime.

**Fig. 6:**
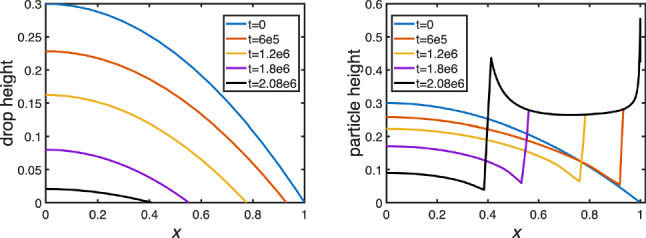
Time evolution of droplet profile and particle height profile, computed with $l_c=10^{-3}$, ${\textrm{Ca}}=10^{-6}$, and $\beta =10^5$

**Fig. 7:**
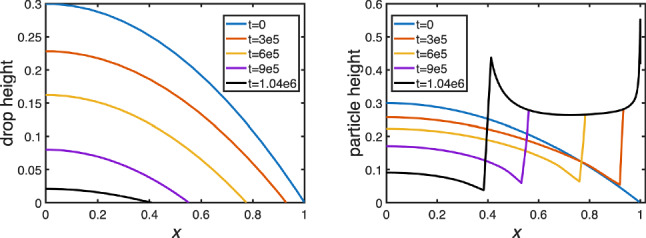
Time evolution of droplet profile and particle height profile, computed with $l_c=10^{-3}$, ${\textrm{Ca}}=2\times 10^{-6}$, and $\beta =5\times 10^4$

Figures 6 and 7 present the time evolution of droplet profile *h*(*x*, *t*) and particle height profile $\psi (x,t)$ computed for $(\textrm{Ca}=10^{-6}, \beta =10^5)$ and $(\textrm{Ca}=2\times 10^{-6}, \beta =5\times 10^4)$, which correspond to weak contact line friction and hence large contact line displacement. The scaling property for the similarity between $(\textrm{Ca}, \beta )$ and $(2\textrm{Ca}, \beta /2)$ is verified by comparing the two evolution processes. The process in Fig. 7 is twice as fast as that in Fig. 6.

**Fig. 8:**
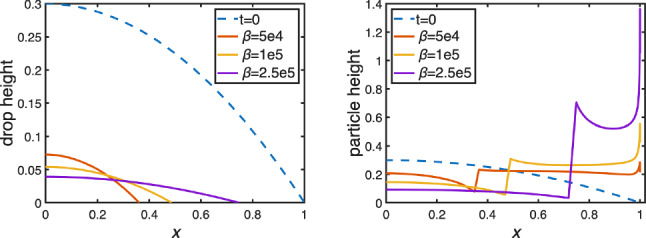
Droplet profile and particle height profile with a volume equaling $\simeq 10\%$ of the initial volume (same below for Figs. 9, 12, and 13), computed with $l_c=10^{-3}$, ${\textrm{Ca}}=10^{-6}$, and three different values of $\beta$

**Fig. 9:**
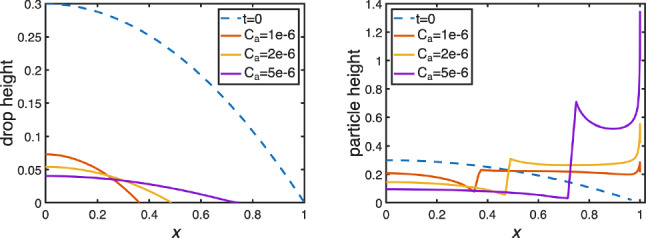
Droplet profile and particle height profile toward the end of drying, computed with $l_c=10^{-3}$, $\beta =5\times 10^4$, and three different values of ${\textrm{Ca}}$

Figures 8 and 9 are produced for *h*(*x*, *t*) and $\psi (x,t)$ by further varying $\beta$ and $\textrm{Ca}$. They all lead to the conclusion that either stronger contact line pinning or faster droplet drying can make the particle deposition more concentrated in a narrower region next to the initial contact line.

#### Transition-limited regime

**Fig. 10:**
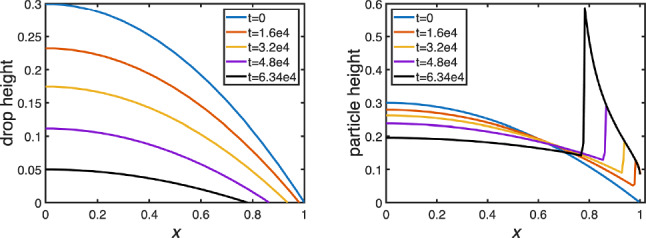
Time evolution of droplet profile and particle height profile, computed with $l_c=10^{3}$, ${\textrm{Ca}}=3\times 10^{-3}$, and $\beta =3\times 10^4$

**Fig. 11:**
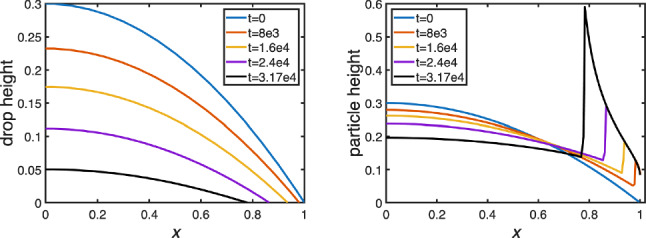
Time evolution of droplet profile and particle height profile, computed with $l_c=10^{3}$, ${\textrm{Ca}}=6\times 10^{-3}$, and $\beta =1.5\times 10^4$

The transition-limited regime with depinned contact lines has been studied by using a reduced model based on OVP [22]. This approach makes use of the homogeneity of the evaporation flux and the linearity of the liquid velocity, as shown in Figs. 2 and 5. Here we perform a numerical investigation in this regime using $l_c=10^3$.

Figures 10 and 11 present the time evolution of droplet profile *h*(*x*, *t*) and particle height profile $\psi (x,t)$ computed for $({\textrm{Ca}}=3\times 10^{-3}, \beta =3\times 10^4)$ and $({\textrm{Ca}}=6\times 10^{-3}, \beta =1.5\times 10^4)$. A comparison between the two evolution processes verifies the scaling property for the similarity between $(\textrm{Ca}, \beta )$ and $(2\textrm{Ca}, \beta /2)$ once again.

**Fig. 12:**
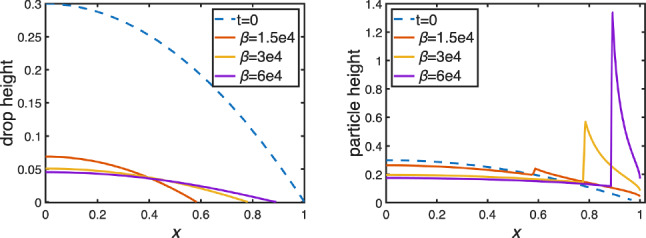
Droplet profile and particle height profile toward the end of drying, computed with $l_c=10^3$, ${\textrm{Ca}}=3\times 10^{-3}$, and three different values of $\beta$

**Fig. 13:**
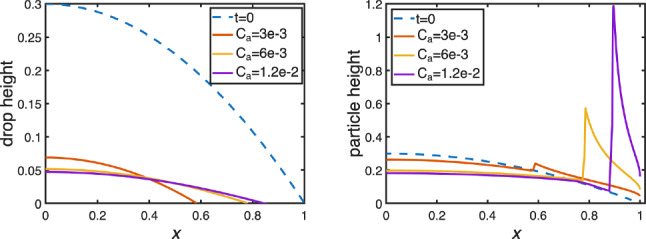
Droplet profile and particle height profile toward the end of drying, computed with $l_c=10^3$, $\beta =1.5\times 10^4$, and three different values of ${\textrm{Ca}}$

Figures 12 and 13 are produced for *h*(*x*, *t*) and $\psi (x,t)$ by varying the contact line friction coefficient $\beta$ and the capillary number $\textrm{Ca}$. Similar to the finding in Sect. 4.3.1, a stronger contact line pinning or faster droplet drying can concentrate the particle deposition into a narrower region. It is noted that the particle height profiles in Figs. 10, 11, 12 and 13 display similar features to those in Ref. [22] (see Fig. 4 there).

## Concluding remarks

In the present work, we have investigated a coupled system that consists of an evolving thin droplet with a moving contact line and a density field of vapor undergoing diffusion in an evolving domain. By employing OVP, we have derived a hydrodynamic model that is comprised of an evolution equation for the droplet profile, a diffusion equation for the vapor density field, a dynamic boundary condition for the contact line velocity, and a constitutive equation for the evaporation flux. Numerical simulations have been carried out to reveal the two distinct regimes of droplet evaporation, namely, the diffusion-limited regime and the transition-limited regime, which can be accessed by tuning one single parameter that is of the length dimensionality and measures the competition between diffusion and evaporation. Capable of accurately computing the liquid velocity in the evolving droplet, we have further simulated the formation of deposit pattern for drying droplets of colloidal suspension.

To conclude we first summarize the major progresses made in this work and then discuss directions for future study. In Ref. [10], evaporation flux and liquid velocity that diverge near the pinned contact line were deduced by taking an analogy with an equivalent electrostatic problem in the diffusion-limited regime where the vapor density is fixed at the saturation density at the drop surface. In Ref. [12], a mathematical model was presented where the droplet is spherical, the contact line is pinned, and the coupled system is in the diffusion-limited regime. In Ref. [22], OVP was employed to derive a reduced model that deals with a spherical droplet with a moving contact line and a homogeneous evaporation flux that corresponds to the transition-limited regime. Compared to these three works, our model can deal with arbitrary droplet shape, which is evolved by the interfacial evaporation and the interfacial tension in the regime of Stokes flow, supplemented with a constitutive equation for the evaporation flux and a dynamic boundary condition for the moving contact line. Neither a spherical or parabolic droplet profile nor a pinned contact line is assumed. Furthermore, a vapor density fixed at the saturation density at drop surface is no longer assumed. Instead, it is obtained as part of the solution in the diffusion-limited regime. We have performed numerical simulations in the diffusion-limited and transition-limited regimes, respectively, where distinct flux distributions at droplet surface have been uncovered, accompanied with different power laws for the amount of solid deposition. In particular, the power-law diverging evaporation flux and liquid velocity near the contact line have been accurately captured. Derived in the framework of OVP, our model is consistent with the gradient dynamics models in Ref. [9] but explicitly incorporates the diffusion equation for vapor transport. It is noted that in Ref. [9], there is also a full model that couples the Stokes equation of liquid and the diffusion equation of vapor.

The variational framework and the numerical scheme can be generalized to investigate the evaporation of binary-mixture liquid droplets [6]. This would involve the free energy of mixing, the Marangoni flow due to surface tension gradient, and the resulting nonspherical droplet shape, which fall within the theoretical framework and numerical capability presented in this work. Since the BEM can be applied to an arbitrary evolving domain, the present approach can be readily generalized to investigate the vapor-mediated interaction in the presence of multiple droplets [43]. It is also of interest to investigate the dynamics of evaporating droplets in the strong-slip regime [55], where the dissipation at the liquid–solid interface, described by an effective Navier slip, is dominant, as experimentally observed in polymer films [56].

## Data Availability

The data that support the findings of this study are available from the corresponding author upon reasonable request.

## Numerical implementation

Following the description in Sect. 3, here we outline the numerical procedure for solving the coupled system in Eq. (18) at each discrete time step. Using the droplet profile $h^{(k)}$ and the contact line position $R^{(k)}$ given at time $t^{(k)}$, we proceed as follows:

*Step 1: Solving the Laplace equation for vapor density.* Given the current interface $z = h^{(k)}(x)$, the gas domain is determined. The Laplace equation $\nabla ^2 c = 0$ is solved in this domain subject to the boundary conditions (17) and the Robin condition $l_c \partial _{\textbf{n}}c = \log (1/c)$ at the interface. The BEM [46] is employed to handle the irregular geometry, yielding the normal derivative $\partial _{\textbf{n}}c^{(k+1)}$ on the interface for updating the droplet profile.

*Step 2: Updating the droplet profile.* Using the newly obtained evaporation flux $\partial _{\textbf{n}}c^{(k+1)}$, the thin film equation is integrated in time by using an explicit Euler scheme. With the coordinate transformation $\xi = x/R^{(k)}$ mapping the moving domain to $[-1,1]$, the updated $h^{(k+1)}$ reads

A1 $$\begin{aligned} \begin{aligned} h^{(k+1)}&= h^{(k)} + \Delta t \Bigg [ - {\textrm{Ca}}\partial _{\textbf{n}}c^{(k+1)} +\frac{\dot{R}^{(k)}\xi }{R^{(k)}}\frac{\partial h^{(k)}}{\partial \xi } \\&\quad - \frac{1}{3R^{(k)}}\frac{\partial }{\partial \xi }\left( \frac{(h^{(k)})^3}{(R^{(k)})^3}\frac{\partial ^3 h^{(k)}}{\partial \xi ^3}\right) \Bigg ], \end{aligned} \end{aligned}$$

with $h^{(k)}(\pm R^{(k)},t^{(k)})=0$. The spatial derivatives are computed using second-order finite differences. Meanwhile, the contact line position is updated via

A2 $$\begin{aligned} R^{(k+1)} = R^{(k)} + \frac{\Delta t}{2\beta }\left[ (\partial _x h^{(k)})^2|_{x=R^{(k)}} - \theta _e^2\right] , \end{aligned}$$

with the slope $(\partial _x h^{(k)})|_{x=R^{(k)}}$ at the contact line approximated by a one-sided difference.

*Step 3: Remeshing if necessary.* The evolution of droplet shape and contact line position is monitored. If the distortion exceeds a predetermined threshold, the gas domain is reconstructed and the BEM mesh is regenerated for the next time step. Otherwise, the existing mesh is reused or simply updated.

The value of *N* used for numerically solving Eq. (19) is $N=200$, with the grid size $\Delta \xi =2/N=0.01$. This grid size is generally believed to be much greater than the length scale at which the no-slip condition breaks down. [For a drop of millimeter scale, $2/N=0.01$ means tens of micrometers, much greater than a typical slip length of nanometer scale.] Subject to the no-slip condition and the grid size as a cut-off length scale, the viscous dissipation due to the thin film evolution is measured by $(1/\theta _d)\eta \ln (R/a)$, which is to be compared to the contact-line friction coefficient $\beta$. Here $\theta _d$ is the dynamic contact angle, $\eta$ is the shear viscosity, and *a* denotes the cut-off length scale, with $R/a\approx 100$. The formation of ring-like deposition patterns necessitates the use of sufficiently large values for $\beta$. In our simulations, we have $(1/\theta _d)\eta \ln (R/a)\sim 100\eta$ and $\beta \sim 10^4\eta$, which make the contact-line slip dominate the total dissipation. By further increasing *N*, $\ln (R/a)$ would only be increased weakly (i.e., logarithmically), and consequently the numerical results are not sensitive to further decrease of grid spacing.

## Scaling laws for particle deposition

Here we derive the power-law growth of the particle deposition amount in each of the two evaporation regimes. The corresponding numerical results are presented in Sect. 4.2.

*Diffusion-limited regime with exponent 4/3*. In this regime, the evaporation flux $J_{\text {ev}}(x)$ diverges in approaching the contact line, taking the form of $J_{\text {ev}}(x) \sim (R - x)^{-1/2}$ for small contact angle. For a quasi-static droplet, the height-averaged velocity near the contact line scales as

A3 $$\begin{aligned} \bar{u}(x) \sim \frac{1}{h(x)} \int _x^R J_{\text {ev}}(s) ds \sim (R - x)^{-1/2}. \end{aligned}$$

Therefore, both $J_{\text {ev}}(x)$ and $\bar{u}(x)$ diverge as $(R - x)^{-1/2}$ near the contact line, as shown in Fig. 3.

For a fluid element initially at $x=x_0$, it takes time $t_d(\delta _0)$ to cover the distance $\delta _0 = R - x_0$ to reach the contact line:

$$ t_d(\delta _0) = \int _{x_0}^{R} \frac{\textrm{d}x}{\bar{u}(x)} \sim (R-x_0)^{3/2} = \delta _0^{3/2}, $$

which gives $\delta _0 \sim \left[ t_d(\delta _0)\right] ^{2/3}$. For a thin droplet, the volume near the contact line scales as $\int _{R-\delta _0}^R h(x) \textrm{d}x \sim \delta _0^2$ for small $\delta _0$. Therefore, the mass of the particles deposited at the pinned contact line grows in time according to the power law $\delta _0^2 \sim \left[ t_d(\delta _0)\right] ^{4/3}$, which is the characteristic signature in the diffusion-limited regime with a pinned contact line [10]. The exponent 4/3 is numerically observed in Fig. 4.

*Transition-limited regime with exponent 2*. In this regime, the evaporation flux $J_{\text {ev}}$ is spatially uniform. The droplet volume decreases at a constant rate $\dot{V}(t) = -2J_{\text {ev}}R<0$. The height-averaged velocity $\bar{u}(x,t)$ satisfies the continuity equation $\partial _t h + \partial _x(h\bar{u}) = -J_{\text {ev}}$. Using this continuity equation, the uniform evaporation flux, the pinned contact line, and the parabolic droplet profile, $\bar{u}(x,t)$ can be obtained as $\bar{u}(x,t) = -\frac{\dot{V}(t)}{3V(t)} x$, which is linear in *x* [22]. Close to the contact line, $\bar{u}$ can be taken as a constant with $x\rightarrow R$. For a fluid element initially at $x=x_0$ close to *R*, it takes time $t_d(\delta _0)$ to cover the distance $\delta _0 = R - x_0$ to reach the contact line, with $t_d(\delta _0)\sim \delta _0$. As a result, the mass of the particles deposited at the pinned contact line grows in time according to the power law $\delta _0^2\sim \left[ t_d(\delta _0)\right] ^2$, which characterizes the transition-limited regime. The exponent 2 is numerically observed in Fig. 5.
